# Leveling up: along-level diffusion tensor imaging in the spinal cord of multiple sclerosis patients

**DOI:** 10.3389/fnimg.2025.1599966

**Published:** 2025-08-11

**Authors:** Atlee A. Witt, Anna J. E. Combes, Grace Sweeney, Logan E. Prock, Delaney Houston, Seth Stubblefield, Colin D. McKnight, Kristin P. O’Grady, Seth A. Smith, Kurt G. Schilling

**Affiliations:** ^1^Vanderbilt University Institute of Imaging Science, Vanderbilt University Medical Center, Nashville, TN, United States; ^2^School of Medicine, Vanderbilt University, Nashville, TN, United States; ^3^NMR Research Unit, Queen Square Multiple Sclerosis Centre, UCL Queen Square Institute of Neurology, University College London, London, United Kingdom; ^4^Department of Radiology and Radiological Sciences, Vanderbilt University Medical Center, Nashville, TN, United States; ^5^Department of Biomedical Engineering, Vanderbilt University, Nashville, TN, United States

**Keywords:** spinal cord, brain, diffusion, MRI, vertebral level, multiple sclerosis, relapsing remitting MS

## Abstract

**Introduction:**

Multiple sclerosis (MS) is a chronic neuroinflammatory disease marked by demyelination and axonal degeneration, processes that can be probed using diffusion tensor imaging (DTI). In the brain, white matter (WM) tractography enables anatomically specific analysis of microstructural changes. However, in the spinal cord (SC), anatomical localization is inherently defined by cervical levels, offering an alternative framework for regional analysis.

**Methods:**

This study employed an along-level approach to assess both microstructural (e.g., fractional anisotropy) and macrostructural (e.g., cross-sectional area) features of the SC in persons with relapsing-remitting MS (pwRRMS) relative to healthy controls (HCs).

**Results:**

Compared to conventional whole-cord averaging, along-level analyses provided enhanced sensitivity to group differences. Detailed segmentation of WM tracts and gray matter (GM) subregions revealed spatially discrete alterations along the cord and within axial cross-sections. Notably, while GM atrophy was associated with clinical disability, microstructural changes did not exhibit significant correlations with disability measures.

**Discussion:**

These findings underscore the utility of level-specific analysis in detecting localized pathology and suggest a refined framework for characterizing SC alterations in MS.

## 1 Introduction

Diffusion tensor imaging (DTI) is a quantitative magnetic resonance imaging (MRI) technique that can reveal important microstructural alterations not otherwise captured by conventional imaging. Diffusion imaging measures the random Brownian motion of water molecules, which is influenced by tissue microstructure, enabling inference of both the magnitude and direction of diffusion along fiber tracts (Kolasa et al., 2019). When applied in the brain and spinal cord (SC), DTI-derived indices can describe tissue microstructure and can serve as a surrogate measure of physiological conditions (Schmierer et al., 2007; Alexander et al., 2007). Diffusion-derived indices include fractional anisotropy (FA), a measure of anisotropic water diffusion, and mean, axial, and radial diffusivities (MD, AD, RD) that represent either average magnitude (MD) or direction (AD/RD) of diffusion. These measures are particularly informative in conditions such as multiple sclerosis (MS), a demyelinating neuroinflammatory condition of the central nervous system. In MS, decreased FA and increased RD are associated with of axonal loss (Sbardella et al., 2013) and myelin injury (Klawiter et al., 2011), respectively, and found in both MS normal-appearing white matter (WM) and lesions compared to healthy control (HC) brains as supported by post-mortem histology (Ciccarelli et al., 2008; Kolasinski et al., 2012; Lin et al., 2007).

Investigations into SC microstructure using diffusion measures can be conducted at multiple spatial scales, encompassing (1) whole-cord metrics, (2) tissue-specific assessments within WM and gray matter (GM), and (3) finer-scale analyses of functionally relevant WM tracts or GM regions. This hierarchical approach has provided key insights, revealing distinct patterns of WM and GM involvement across MS subtypes, with lesion burden varying across tissue classes (Eden et al., 2019; Ouellette et al., 2020; Kreiter et al., 2024). Expanding this framework, recent studies have examined specific WM pathways and GM subregions, such as the lateral funiculi, where lesion burden has been correlated with clinical disability scores, including the Expanded Disability Status Scale (EDSS) (Eden et al., 2019). However, it remains unclear which spatial scale – whole cord, tissue compartment, or individual pathways – offers the most sensitive biomarker for distinguishing persons with MS (pwMS) from HCs or for evaluating relationships between microstructural integrity and clinical impairment.

Despite their utility, diffusion-derived indices are often averaged across large swaths of tissue, potentially obscuring localized microstructural alterations (Tallus et al., 2023). Tractography is one such attempt to more directly model WM structural integrity and localize variations in diffusion along a specific fiber pathway (Colby et al., 2012; Pieri et al., 2021). In the SC, however, such along-structure analysis does not require tractography, as the anatomical localization is inherently defined by cervical levels. This level-based approach enables a lengthwise parcellation of diffusion data, capturing regional variations along the cord from, for example, C2 through C5, without extensive post-processing demands. Prior studies have leveraged level-based atlases to understand the distribution of MS lesions along the corticospinal tract from the cortex to the cervical SC, highlighting the feasibility to derive anatomic details via atlas (Kerbrat et al., 2020). An atlas-based approach enables a comprehensive investigation of structural changes – both within and along the SC – while also revealing the spatial scale at which these changes occur.

In addition to microstructure, tissue macrostructure measures have proven useful in distinguishing diseased and healthy SCs. For example, macrostructural features like SC atrophy have been identified as potential predictors of subclinical disease progression and an increased risk of conversion from relapsing disease to secondary progressive disease (Bischof et al., 2022). Cross-sectional area (CSA) is a proxy measure for SC atrophy, with an underexplored opportunity to derive CSA across finer SC levels compared to the conventional approach across multiple slices limited to C2 and C3 vertebral levels (Keegan et al., 2024). A prior study assessing SC atrophy across vertebral levels found atrophy to be evenly distributed, with no apparent correlation between lesion distribution and atrophy (Bussas et al., 2022). Moreover, no association was observed between atrophy and/or lesion distribution with disability, including EDSS and sensorimotor tests (SMT) like the Timed 25-Foot Walk (T25). However, to our knowledge, these findings have not been examined in the context of diffusion-derived indices, nor has atrophy been systematically evaluated in relation to lesion burden across specific WM pathways and GM subregions.

In this study, we sought to investigate differences in macrostructural and microstructural SC integrity between HC and pwRRMS via an along-level approach and across multiple spatial scales. We also set out to associate these findings with disability. By identifying specific locations along the SC where disease-related alterations are most pronounced, we aim to refine MS biomarkers and improve our understanding of the spatial distribution of neurodegenerative processes in the human SC.

## 2 Methods

### 2.1 Image acquisition

Collection of anatomical and DTI data was approved by the Vanderbilt Institutional Review Board Health Sciences Committee and performed in accordance with relevant ethical guidelines and regulations. 70 pwRRMS and 46 HCs were recruited to undergo SMT and cervical SC MRI protocol at 3 T. Inclusion criteria for patients included EDSS score of less than 4, relapsing–remitting disease, and no contraindications to 3 T MRI (Kurtzke, 1983). Subjects were asked to perform SMT, including the Timed Up and Go (TUG) test (Sebastião et al., 2016) and T25. Participants were then scanned on a 3 T Philips Elition X (Philips Medical Systems, Best, The Netherlands) using a dual-channel transmit body coil and 16-channel neurovascular coil for signal reception centered in the cervical SC at C3 to C4 to encompass C2-C5 cervical vertebral levels.

The acquisition protocol included:

- Sagittal T2-weighted turbo spin echo (TR/TE = 2500/100 ms, *α* = 90°, FOV = 160 × 250 mm^2^, 18 slices, voxel size 0.8 × 1 × 2 mm^3^)
- Multi-echo Fast Field Echo (mFFE) T2*-weighted axial anatomical scan (TR/TE = 700/8 ms, α = 28°, FOV = 160 × 160 mm^2^, 14 slices, voxel size 0.65 × 0.65 × 5 mm^3^)
- Cardiac-triggered single-shot EPI diffusion sequence (TR = 5 beats (∼4,000 ms), TE = 77 ms, SENSE (RL) = 1.8, FOV = 80 × 57.5 × 70 mm^3^, 14 slices, resolution = 1.1 × 1.1 × 5 mm^3^, averages = 3) with a single shell acquisition (15 directions, b = 750 s/mm^2^)

### 2.2 Image processing

The image processing steps were adapted from prior work and utilized the Spinal Cord Toolbox (SCT) and FSL (De Leener et al., 2017; Barry et al., 2016; Combes et al., 2022; Smith et al., 2004; Jenkinson et al., 2012). For each participant, vertebral levels were identified on the sagittal T2 image using SCT and registered to the mFFE image. Cord and GM segmentations were obtained on the mFFE image using *sct_deepseg_sc* (Gros et al., 2019) and *sct_deepseg_gm* (Perone et al., 2018), respectively. The WM mask was obtained by subtracting the GM mask from the full cord mask obtained via the initial segmentation. Unsatisfactory segmentations were corrected manually. Lesions were identified and masked on the T2*-weighted mFFE scan by two trained neuroradiologists (CM and SS) using ITK-Snap. For all further analyses, the whole cord (mFFE), WM, and GM regions were masked as the entire region, whether or not a lesion was present. The cord, WM, GM, and lesion masks were then resampled to atlas resolution (0.5 × 0.5 × 0.5 mm^3^). Per-slice CSA was calculated via the SCT command *sct_process_segmentation* (Valošek et al., 2024) from vertebral level C2 through C5 for the cord, WM, and GM masks.

Diffusion data underwent denoising via the Marcenko-Pastur PCA algorithm (Tournier et al., 2019; Cordero-Grande et al., 2019; Veraart et al., 2016a; Veraart et al., 2016b), Gibbs artifact removal (Tournier et al., 2019; Kellner et al., 2016), and Rician bias noise removal (Tournier et al., 2019) prior to motion correction using *sct_dmri_moco* (Cordero-Grande et al., 2019; Veraart et al., 2016a). Model fitting was performed using SCT to obtain FA, AD, RD, and MD maps which were then transformed to the anatomical space and resampled to atlas resolution (0.5 × 0.5 × 0.5 mm^3^).

To identify vertebral levels, the PAM50 continuous level map from SCT was first transformed to anatomical space as defined by the mFFE image from each subject. Then, to derive masks for six WM pathways, the PAM50 WM atlas was used to produce binary masks for dorsal, lateral, and ventral columns in the left and right hemicords [left dorsal (LDo), right dorsal (RDo), left ventral (LV), right ventral (RV), left lateral (LL), right lateral (RL)]. A similar process was repeated to derive masks for six GM subregions including the dorsal, intermediate, and ventral horns in the left and right hemicords [left dorsal horn (LDH), right dorsal horn (RDH), left ventral horn (LVH), right ventral horn (RVH), left intermediate horn (LIH), right intermediate horn (RIH)]. The WM pathway and GM subregion masks were transformed to anatomical space, resampled to atlas resolution (0.5 × 0.5 × 0.5 mm^3^), and binarized above 0.5. Per-slice CSA was calculated via the SCT command *sct_process_segmentation* from vertebral level C2 through C5 for WM pathway and GM subregion data.

### 2.3 Extraction of image metrics

Participants were excluded from the study due to missing data or poor data quality including MS subjects with no EDSS or SMT scores recorded (*n* = 5), poor vertebral level labeling that could not be manually corrected (*n* = 1), or no generation of lesion masks (*n* = 10). After removal of subjects, analysis was performed on 46 HCs (31.9 +/− 6.8 years old, 29 females) and 54 pwRRMS (36.7 +/− 7.5 years old, 36 females, EDSS (1 [median], 0–3.5 [range])).

Image analysis proceeded via in-house MATLAB code (R2024a). The PAM50 continuous level mask was extrapolated to a new mask with discrete, ascending increments of 0.2 (i.e., C2.0, C2.2, C2.4…) until C5.8. Then, for both HC and pwRRMS, per-slice CSA was extracted for each discrete sub-level within anatomical masks including whole cord, WM, GM, and WM pathway/GM subregion masks. C6 refers to the superior aspect of C6 and the inferior aspect of C5 but does not encompass C6 as this was not fully included in the original scan acquisition. Diffusion-derived indices were extracted (FA, AD, RD MD) using masks at each discrete vertebral sub-level. Finally, for just pwRRMS, lesion load was calculated as the volume of lesion divided by the volume of the whole cord, WM, or GM mask across vertebral levels.

**Figure 1:**
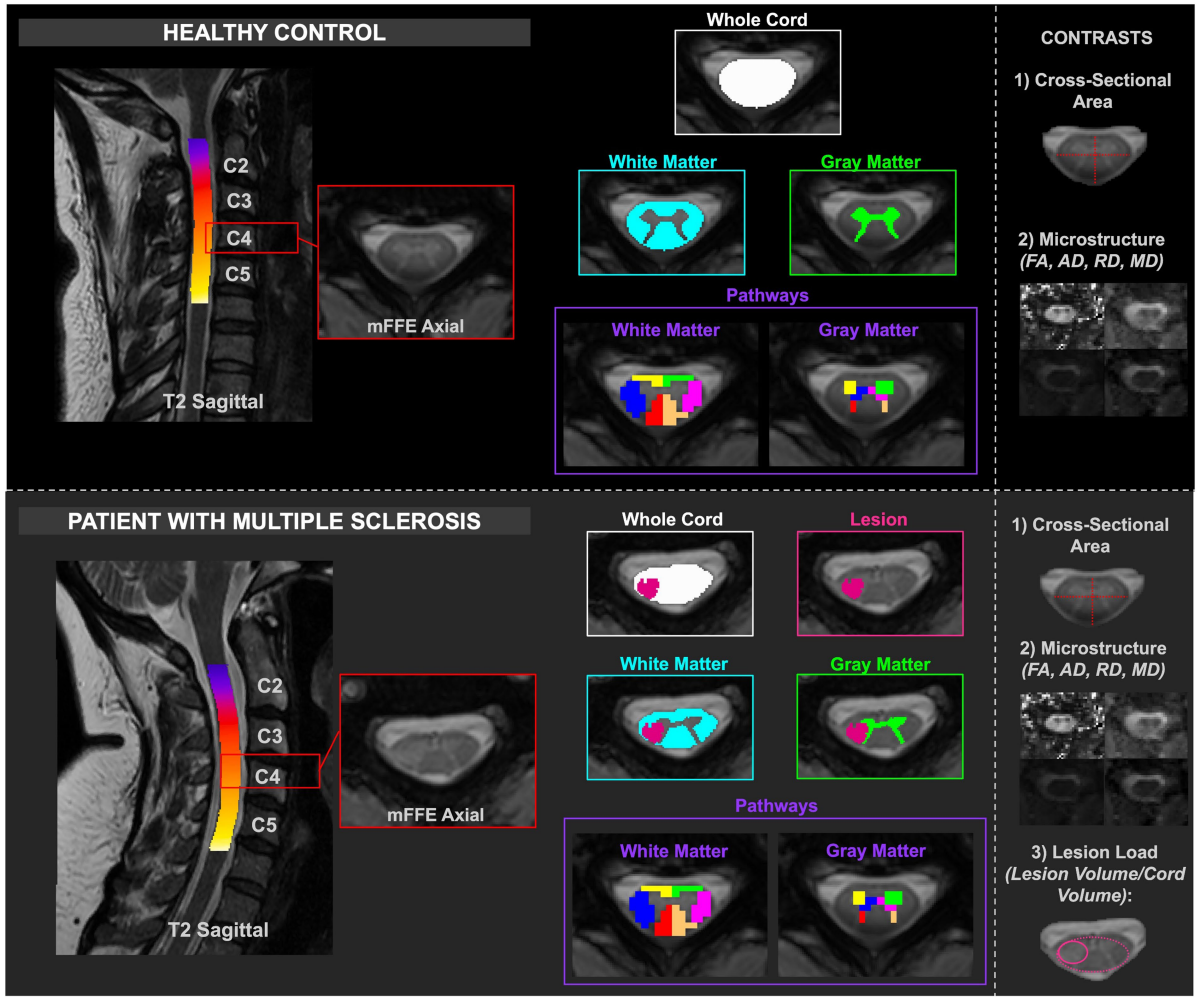
Depiction of healthy and MS cord processing, including delineation of the masks relative to healthy or lesioned tissue. The contrasts are included in the right column. CSA and diffusion-derived indices were calculated for HCs, and CSA, diffusion-derived indices, and lesion load were calculated for pwRRMS.

Figure 1 summarizes the data analysis approach, including assessing macrostructure and microstructure features derived from (1) the whole cord, (2) specific tissues (WM/GM), and (3) regions-of-interests in both HCs and pwRRMS. For pwRRMS, lesion load was derived for whole cord and WM/GM analyses.

### 2.4 Statistical analysis

Group differences in age between HCs and pwRRMS were assessed via Welch’s *t*-test, while differences in sex distribution were evaluated using a chi-squared analysis. Clinical outcomes, including TUG and T25, were compared using ANCOVA adjusting for age and sex.

Meam values for CSA, diffusion-derived indices, and lesion load were determined between C2 through C5 for HC and pwRRMS for each anatomic mask (whole cord, WM, and GM), with significance determined via Welch’s t-test as *p < 0.05*. Group-averaged means and 95% confidence intervals were plotted across levels for CSA and diffusion-derived indices. Because the same subjects contribute measurements at equidistant levels in 0.2 increments, a linear mixed-effect model was appied at each level. The model included fixed effects for group (HC vs. pwRRMS), spinal level, and their interaction, with subject included as a random intercept: Imaging Feature ~ Group * Level + (1 | Subject). Significance was defined as *p < 0.01.*

For WM and GM pathways, Cohen’s d effect size differences were calculated for CSA and diffusion-derived indices, respectively, between HC and pwRRMS by calculating the difference in cohort means divided by the pooled standard deviation; this method was selected as a way to standardize the difference in means for various MRI measures between two groups. Mean values for CSA and diffusion-derived indices were computed per pathway, and between-group differences were assessed using Welch’s *t-*test with a relatively stringent *p < 0.01* to account for tests from multiple pathways (6 WM/6 GM). As above, a linear mixed-effect model was appied at each level for each pathway: Imaging Feature ~ Group * Level + (1 | Subject). Significance was defined as *p < 0.01*.

Multivariable linear regression was performed to understand if CSA or diffusion-derived indices relate to disability as measured by T25, TUG, or EDSS while adjusting for age, sex, and diagnosis as covariates. Continuous variables – including CSA, diffusion-derived indices, and SMT measures – were normalized to allow for a comparable interpretation of effect size across features of different scales. The variables were inputted into MATLAB as *fitlm (predictors, Clinical Feature ~ Imaging Feature + Age + Sex + Diagnosis)*. Effect size (B1) was used to measure the association between the imaging and clinical features. False discovery rate (FDR) *p*-value correction was performed with significance determined as *p < 0.05*. For visualization, effect sizes are normalized between −1 and 1 for macrostructure and microstructure analyses and between −0.5 and 0.5 for disability analyses.

## 3 Results

### 3.1 Group comparisons

Demographic data and clinical variables are reported in Table 1. PwRRMS demonstrated significantly longer TUG and T25 times compared to HCs (both *p < 0.01*). The patient cohort was also significantly older than the HC cohort (*p < 0.01*) with an average age of 36.70 compared to 31.86 in the HC cohort.

**Table 1:** Demographic and clinical averages with standard deviation for healthy controls (HCs) and persons with relapsing–remitting multiple sclerosis (pwRRMS).

	Healthy controls (*n* = 46)	Persons with multiple sclerosis (*n* = 54)	*P*-value (*p*)
Sex at birth	29F/17M	36F/18M	**NS**
Age (years)	31.86 +/− 6.78	36.70 +/− 7.51	<0.01*
Disease duration (years)	–	5.70 (0.1–20.0)	
EDSS (median and range)	–	1 (0–3.5)	
TUG (s)	6.10 +/− 0.91	7.34 +/− 1.81	<0.01*
T25 (s)	4.25 +/− 0.62	4.86 +/− 1.17	< 0.01*

Age was compared using Welch’s t-test. Sex was compared using a chi-squared analysis. TUG and T25 were compared using ANCOVA adjusted for age and sex. EDSS = Expanded Disability Status Scale score; NS = not significant; TUG = Timed Up and Go; T25 = Timed 25-Foot Walk. The bold value means * *p* < 0.01.

### 3.2 Macrostructure, microstructure, and lesion load: whole cord

As listed above, measures related to macrostructure (CSA), microstructure (diffusion-derived indices), and lesion load were obtained from whole cord, WM, and GM masks. In this context, “whole cord” refers to combined WM and GM tissue, while excluding the spinal canal and CSF.

**Figure 2:**
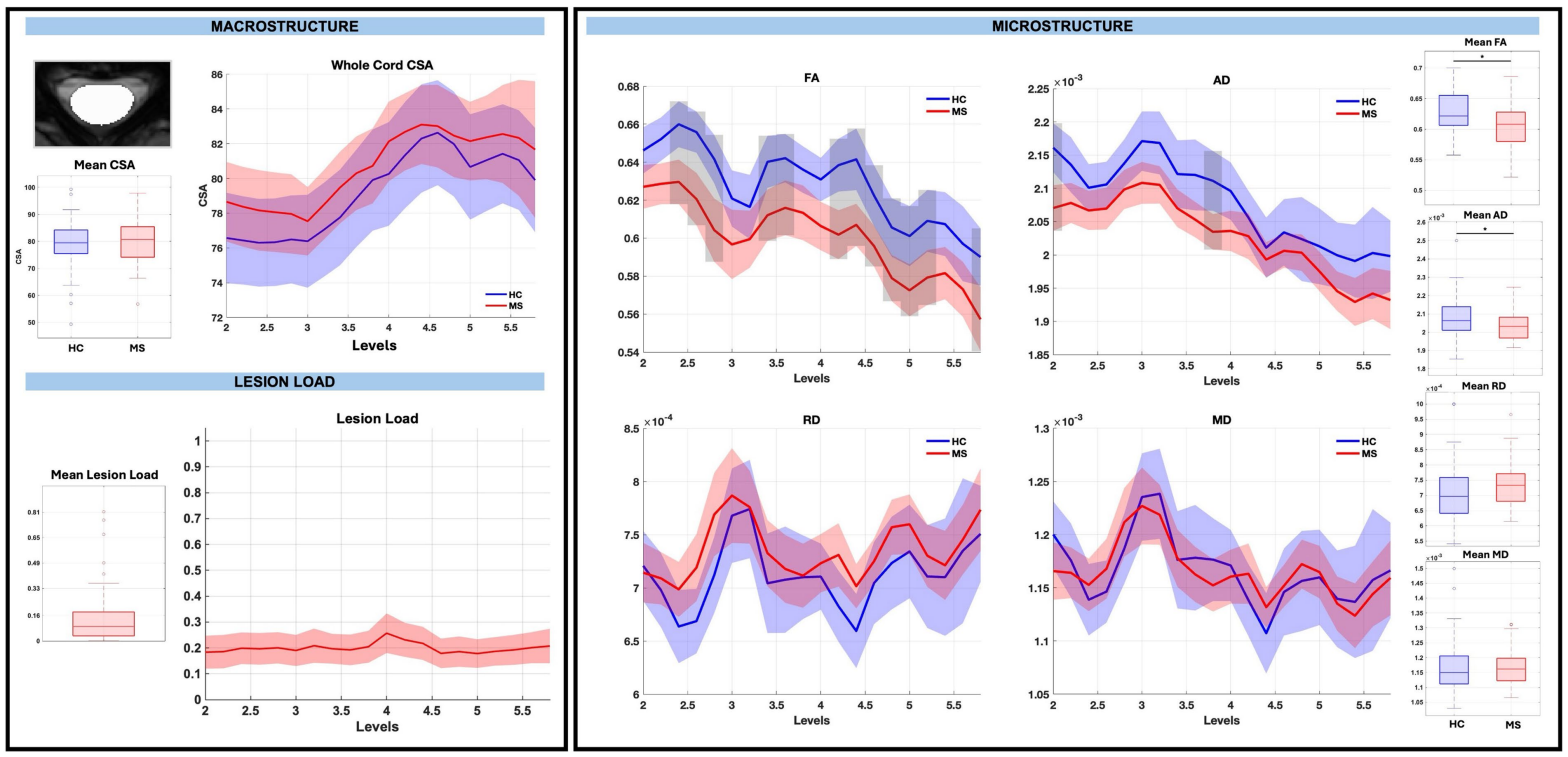
Macrostructure, lesion load, and microstructure for the whole cord mask, including both group average differences and along-level differences. HCs are listed in blue and pwRRMS are listed in red, with the thick lines representing mean values and the shaded regions as 95% confidence intervals. For group differences, the “*” represents significance at *p* < 0.05 as determined by Welch’s t-test. For along-level differences, the gray bars represent a significant difference at that level at *p* < 0.01 as determined by the linear mixed-effect model.

Across the whole cord measures, CSA was not significantly different between pwRRMS and HCs, while microstructure measures showed statistically significant decreases in FA and AD in pwRRMS compared to HCs. There were no significant differences in average whole cord CSA between pwRRMS and HCs, which held true across discrete levels. Average whole cord FA was significantly decreased in pwRRMS (0.60) compared to HCs (0.63) (*p < 0.01*) with specific significant differences localized to sub-levels C2.4 through C2.8 (*p < 0.01*), C3.4 through C3.6 (*p < 0.01*). C4.2 through C5.2 (*p < 0.01*), and C5.8 (*p < 0.01*), suggesting a spatially heterogeneous pattern of microstructural alterations. Average whole cord AD was also significantly decreased in pwRRMS (2.03×10^−3^ mm^2^/s) compared to HCs (2.08×10^−3^ mm^2^/s) (*p = 0.01*), with significant differences localized to sub-levels C2.0 (*p < 0.01*) and C3.8 (*p < 0.01*). Average whole cord RD was greater in pwRRMS (7.35×10^−4^ mm^2^/s) compared to HCs (7.13×10^−4^ mm^2^/s) and MD decreased in pwRRMS (1.16×10^−3^ mm^2^/s) compared to HCs (1.17×10^−3^ mm^2^/s) though neither were significant, with similar findings across levels as shown in Figure 2.

### 3.3 Macrostructure, microstructure, and lesion load: WM and GM

**Figure 3:**
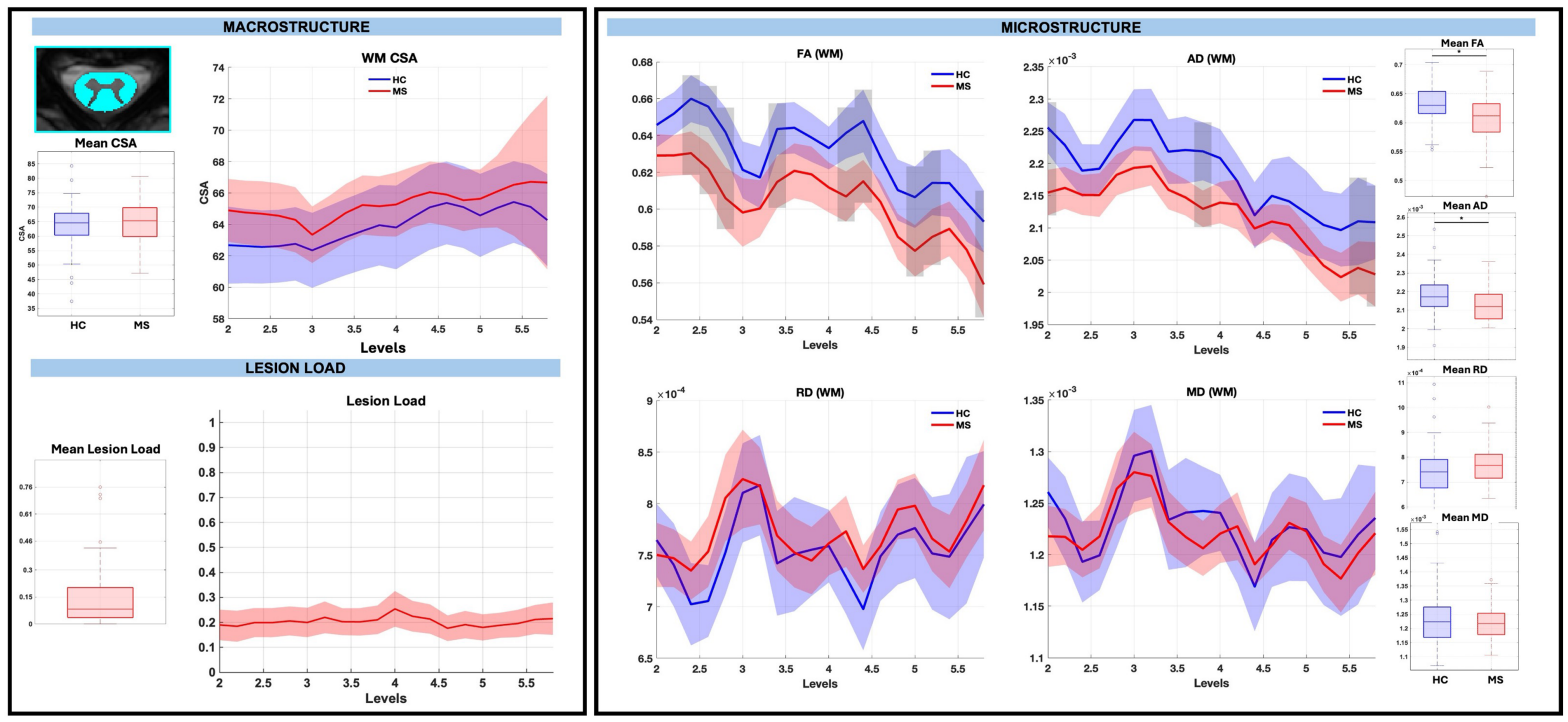
Macrostructure, lesion load, and microstructure for the WM mask, including both group average differences and along-level differences. HCs are listed in blue and pwRRMS are listed in red, with the thick lines representing mean values and the shaded regions as 95% confidence intervals. For group differences, the “*” represents significance at *p* < 0.05 as determined by Welch’s t-test. For along-level differences, the gray bars represent a significant difference at that level at *p* < 0.01 as determined by the linear mixed-effect model.

**Figure 4:**
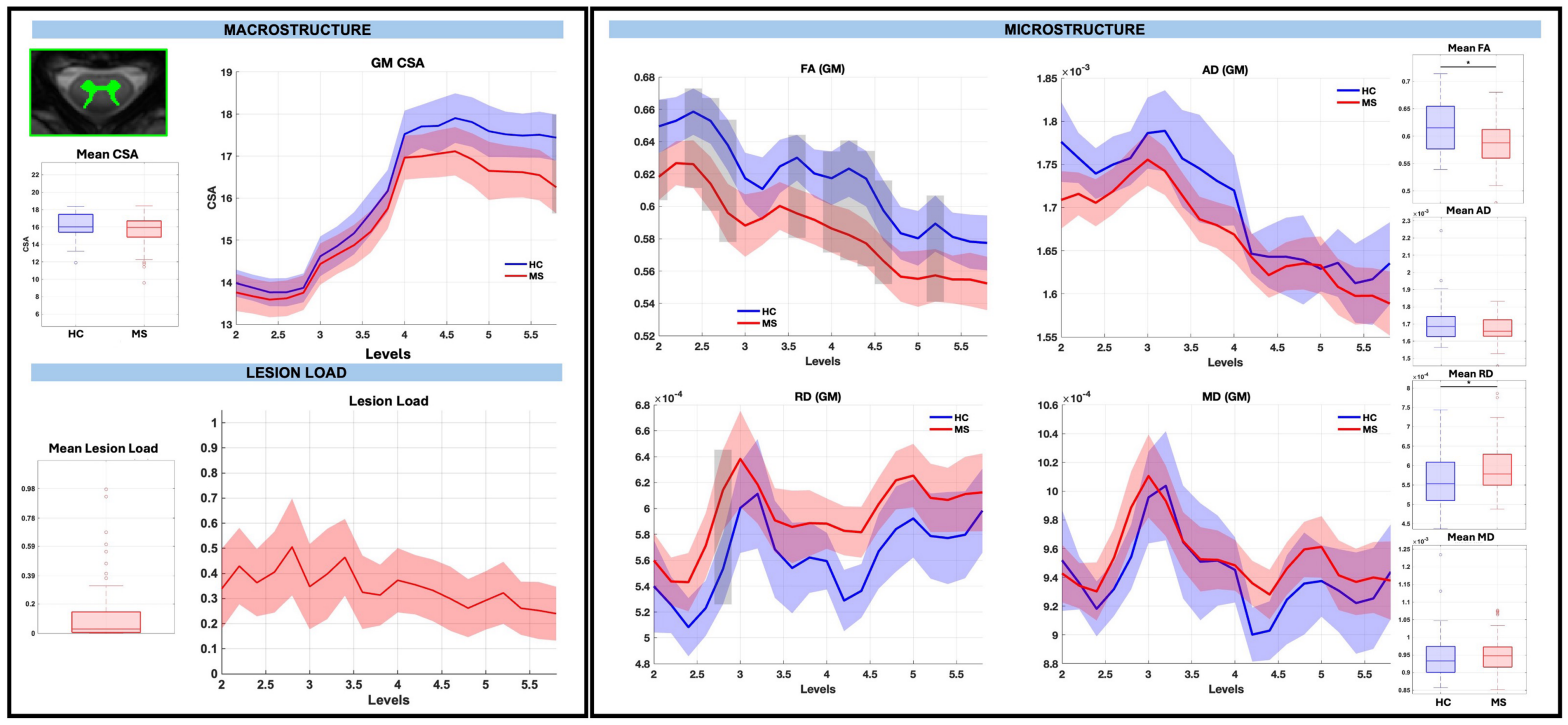
Macrostructure, lesion load, and microstructure for the GM mask, including both group average differences and along-level differences. HCs are listed in blue and pwRRMS are listed in red, with the thick lines representing mean values and the shaded regions as 95% confidence intervals. For group differences, the “*” represents significance at *p* < 0.05 as determined by Welch’s t-test. For along-level differences, the gray bars represent a significant difference at that level at *p* < 0.01 as determined by the linear mixed-effect model.

GM CSA was largely similar between groups, with the exception of a significant reduction at sub-level C5.8 in pwRRMS (*p < 0.01*), while microstructure demonstrated statistically significant decreases in WM and GM FA, decreases in WM AD, and increases in GM RD in pwRRMS compared to HCs. For average WM and GM diffusion-derived indices, FA was significantly decreased in pwRRMS (WM 0.61, GM 0.58) compared to HCs (WM 0.63, GM 0.62) (WM *p < 0.01*, GM *p < 0.01*), AD was significantly decreased in pwRRMS (WM 2.12×10^−3^ mm^2^/s) compared to HCs (WM 2.18×10^−3^ mm^2^/s) (*p < 0.01*), and RD was significantly increased in pwRRMS (GM 5.95×10^−4^ mm^2^/s) compared to HCs (GM 5.63×10^−4^ mm^2^/s) (*p = 0.02*). Specific significant differences across levels are depicted in Figure 3 for WM and Figure 4 for GM. Average lesion loads for whole cord, WM, and GM were 0.200, 0.203, and 0.343, respectively.

### 3.4 Macrostructure, microstructure, and lesion load: WM pathways

There were no significant group differences in CSA in any of the WM pathways, nor were there significant differences for averaged WM RD and MD diffusion-derived indices. WM FA was significantly decreased for RDo (0.64, *p < 0.01*) and LV (0.55, *p < 0.01*) and WM AD was significantly decreased for LL (0.57 mm^2^/s, *p < 0.01*) in pwRRMS compared to HCs.

**Figure 5:**
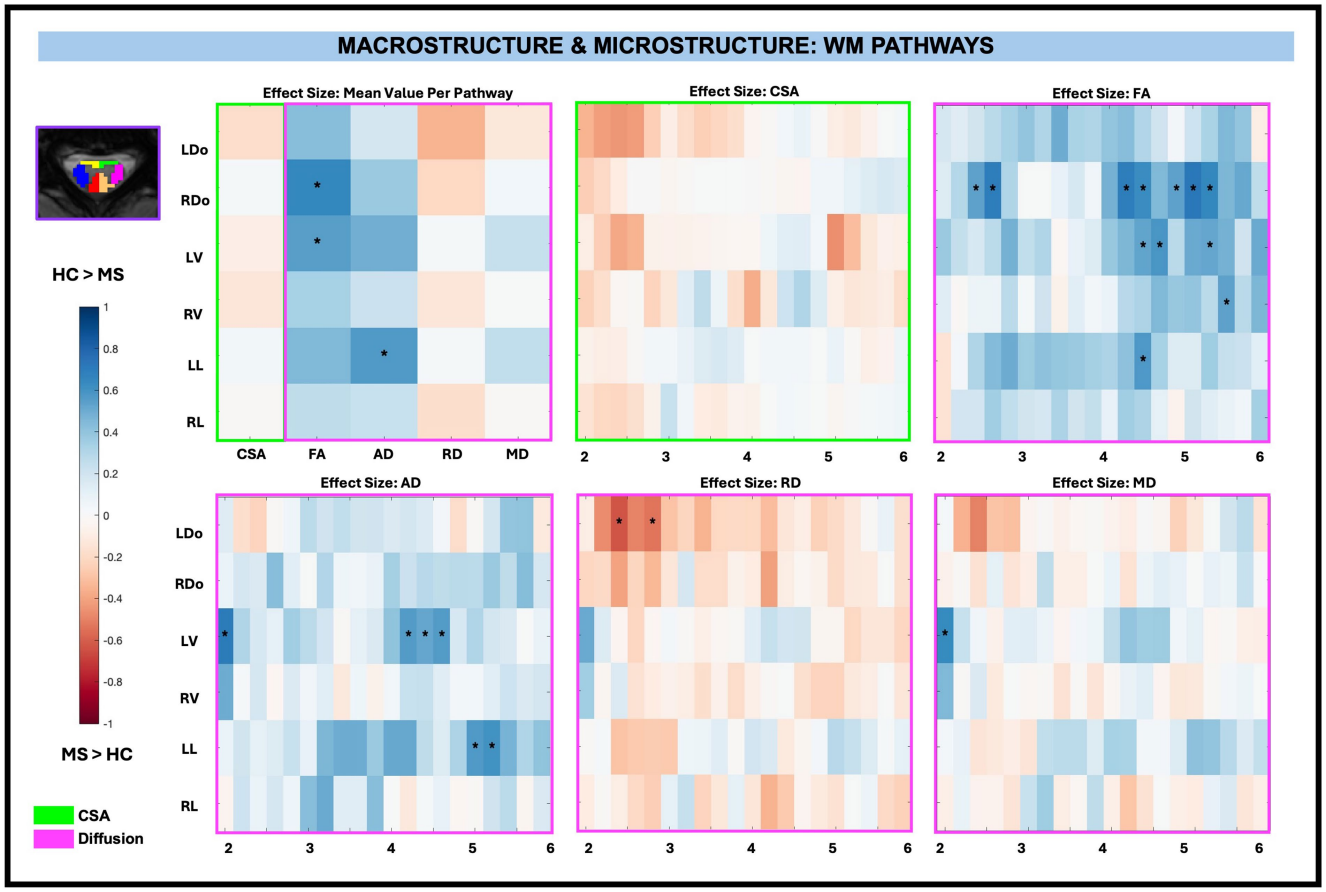
Cohen’s d effect size visualization of WM pathways split into group average differences (left graph) and along-level differences for CSA, FA, AD, RD, and MD. The group differences in CSA (green box) and microstructure (pink box) are indicated for the along-level analyses. Blue squares indicate a decreased effect size in pwRRMS versus HCs and red squares indicate a greater effect size in pwRRMS versus HCs. Significance is denoted with “*” and was determined for the mean values as *p* < 0.01 via Welch’s t-test and for the along-level differences as *p* < 0.01 via the linear mixed-effect model.

There were no significant CSA effect size differences between pwRRMS and HCs across vertebral levels. When considering across-level comparisons, WM FA was significantly decreased for RDo (7 sub-levels), LV (3 sub-levels), RV (1 sub-level), and LL (1 sub-level), AD significantly decreased for LV (4 sub-levels) and LL (2 sub-levels), and MD significantly decreased for LV (1 sub-level) in pwRRMS compared to HCs (*p < 0.01*). WM RD was significantly increased for LDo (2 sub-levels) in pwRRMS compared to HCs (*p < 0.01*). Detailed effect sizes are depicted in Figure 5.

### 3.5 Macrostructure, microstructure, and lesion load: GM subregions

Similar to WM pathways, there were no significant differences in CSA between pwRRMS and HCs for averaged GM subregions, nor were there significant differences for averaged GM AD, RD, and MD diffusion-derived indices. GM FA was significantly decreased for LVH (0.37, *p < 0.01*), RVH (0.74, *p < 0.01*), and LIH (0.69, *p < 0.01*) in pwRRMS compared to HCs.

**Figure 6:**
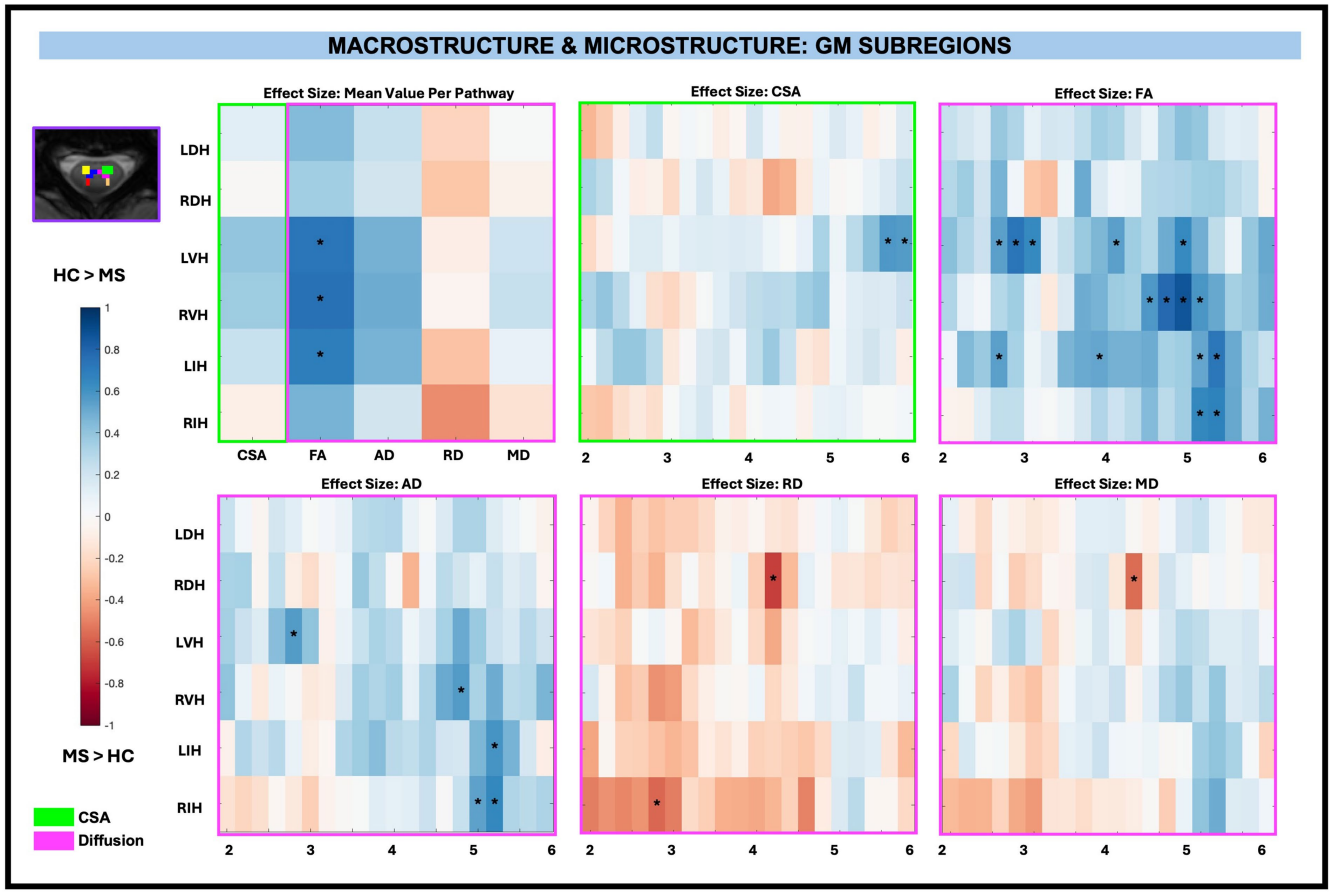
Cohen’s d effect size visualization of GM subregions split into group average differences (left graph) and along-level differences for CSA, FA, AD, RD, and MD. The group differences in CSA (green box) and microstructure (pink box) are indicated for the along-level analyses. Blue squares indicate a decreased effect size in pwRRMS versus HCs and red squares indicate a greater effect size in pwRRMS versus HCs. Significance is denoted with “*” and was determined for the mean values as *p* < 0.01 via Welch’s *t*-test and for the along-level differences as *p* < 0.01 via the linear mixed-effect model.

Across-level comparisons for GM subregions revealed a significant reduction in CSA for LVH (2 sub-levels) in pwRRMS compared to HCs (*p < 0.01*). GM FA was significantly decreased for LVH (5 sub-levels), RVH (4 sub-levels), LIH (4 sub-levels), and RIH (2 sub-levels) and AD significantly decreased for LVH (1 sub-level), RVH (1 sub-level), LIH (1 sub-level), and RIH (2 sub-levels) in pwRRMS compared to HCs (*p < 0.01*). Conversely, GM RD was significantly increased for RDH (1 sub-level) and RIH (1 sub-level) and MD significantly increased for RDH (1 sub-level) in pwRRMS compared to HCs (*p < 0.01*). Detailed effect sizes are depicted in Figure 6.

In summary, no significant differences in CSA were observed between pwRRMS and HCs across averaged regions. However, significant reductions in FA were identified in both WM pathways and GM subregions, along with a decrease in AD specifically in WM pathways. The along-level analysis revealed spatially localized microstructural alterations, with pwRRMS exhibiting lower FA and AD, as well as increased RD, across multiple levels in both WM pathways and GM subregions.

### 3.6 Macrostructure and clinical variables

**Figure 7:**
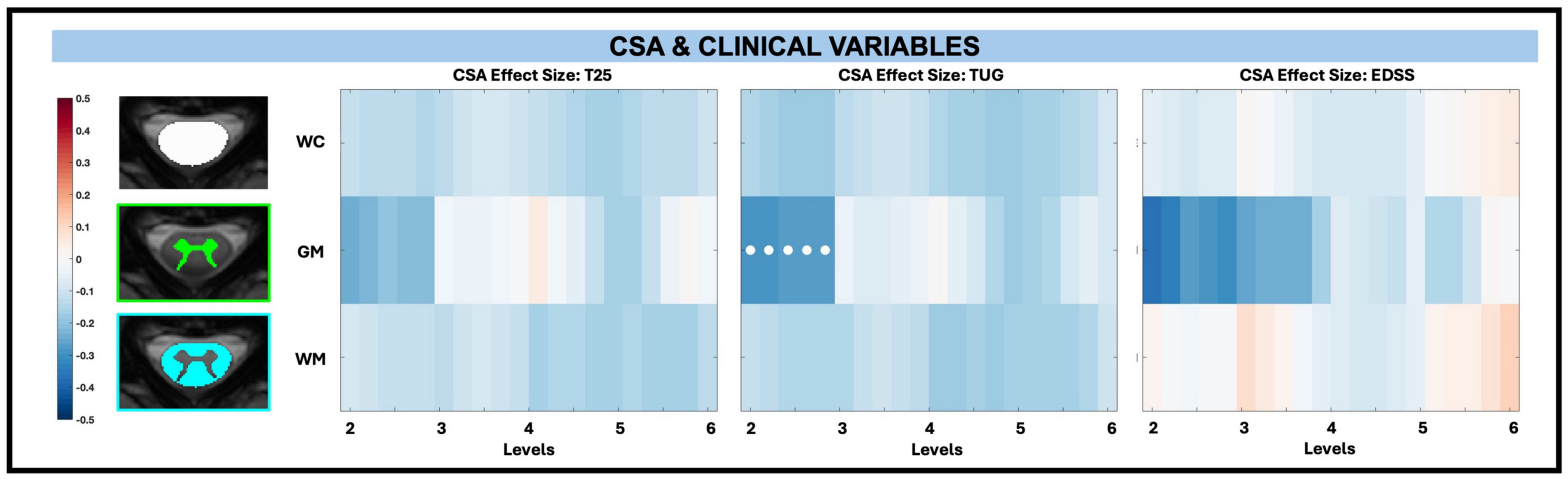
Visualization of linear regression investigating the relationship between CSA and T25, TUG, and EDSS for whole cord (WC), GM, and WM masks. Positive relationships are indicated in red, while negative relationships are indicated in blue. Significant differences are denoted via a white circle, with *p* < 0.05 following FDR correction.

Among macrostructural measures (whole cord, WM, GM, and subregions), only GM CSA exhibited significant associations with clinical disability measures, specifically at C2 to C3. When examining the relationship between averaged CSA and disability measures (T25, TUG, and EDSS) across levels, superior levels (C2 to C3) exhibited the greatest negative effect size (beta values from normalized continuous variables), particularly for GM. After C3, the effect size differences in GM were less pronounced. Decreased CSA was significantly related to increased TUG times for GM between C2 to C3 (beta −0.28, −0.29, −0.27, −0.28, −0.28; *adjusted p = 0.01*) as shown in Figure 7. There were also reductions in CSA with increasing T25 times and increasing EDSS score in the range of C2 to C3, though these findings were not significant after FDR correction.

**Figure 8:**
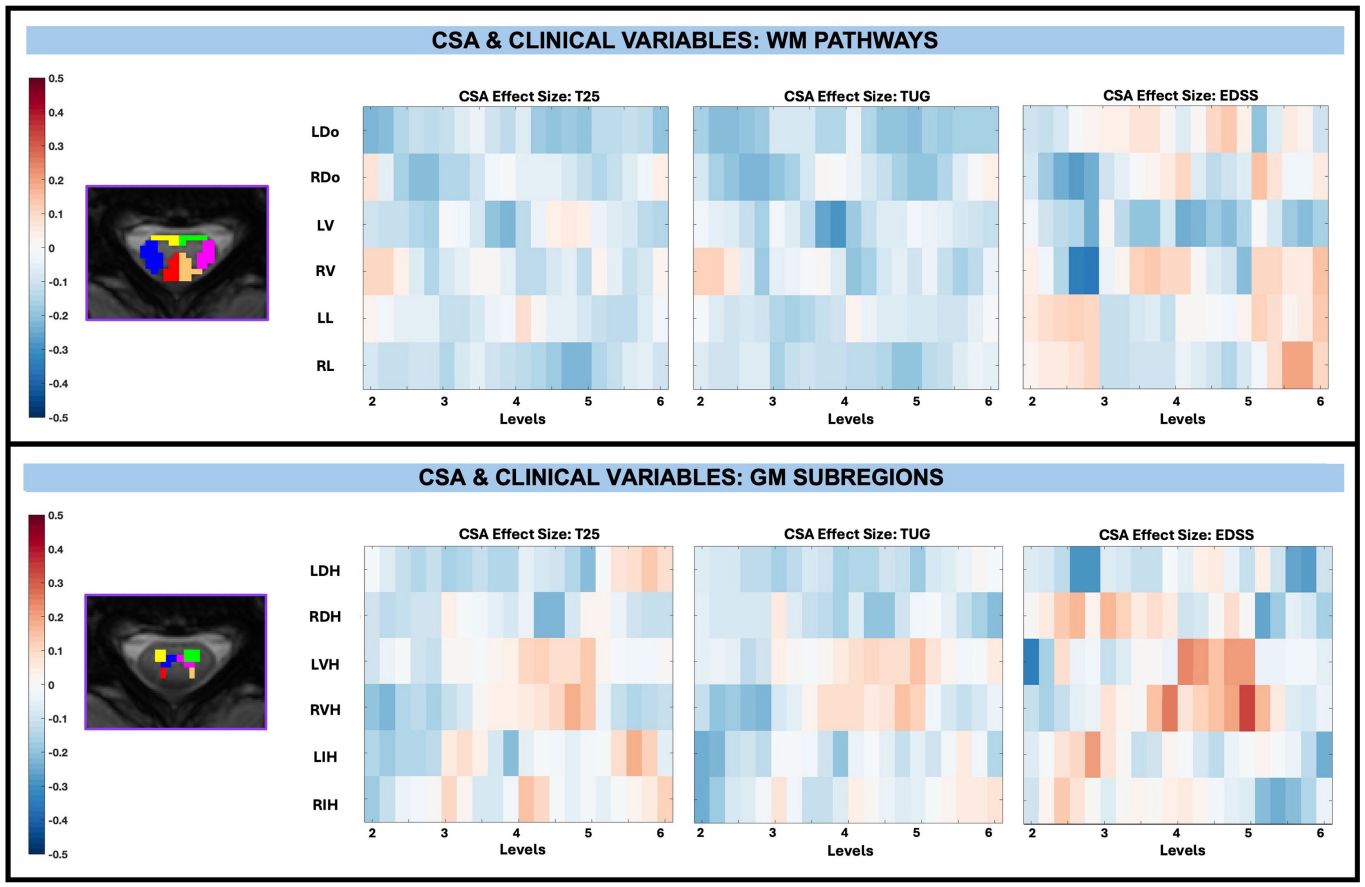
Visualization of linear regression investigating the relationship between CSA and T25, TUG, and EDSS for WM pathways (top) and GM subregions (bottom). Pathways for WM include left dorsal (LDo), right dorsal (RDo), left ventral (LV), right ventral (RV), left lateral (LL), and right lateral (RL). Subregions for GM include left dorsal horn (LDH), right dorsal horn (RDH), left ventral horn (LVH), right ventral horn (RVH), left intermediate horn (LIH), and right intermediate horn (RIH). Positive relationships are indicated in red, while negative relationships are indicated in blue. Significant differences are denoted via a white circle, with *p* < 0.05 following FDR correction.

For CSA and its relationship to disability across levels for WM pathways and GM subregions, there were no significant associations after performing FDR correction as shown in Figure 8.

### 3.7 Microstructure and clinical variables

**Figure 9:**
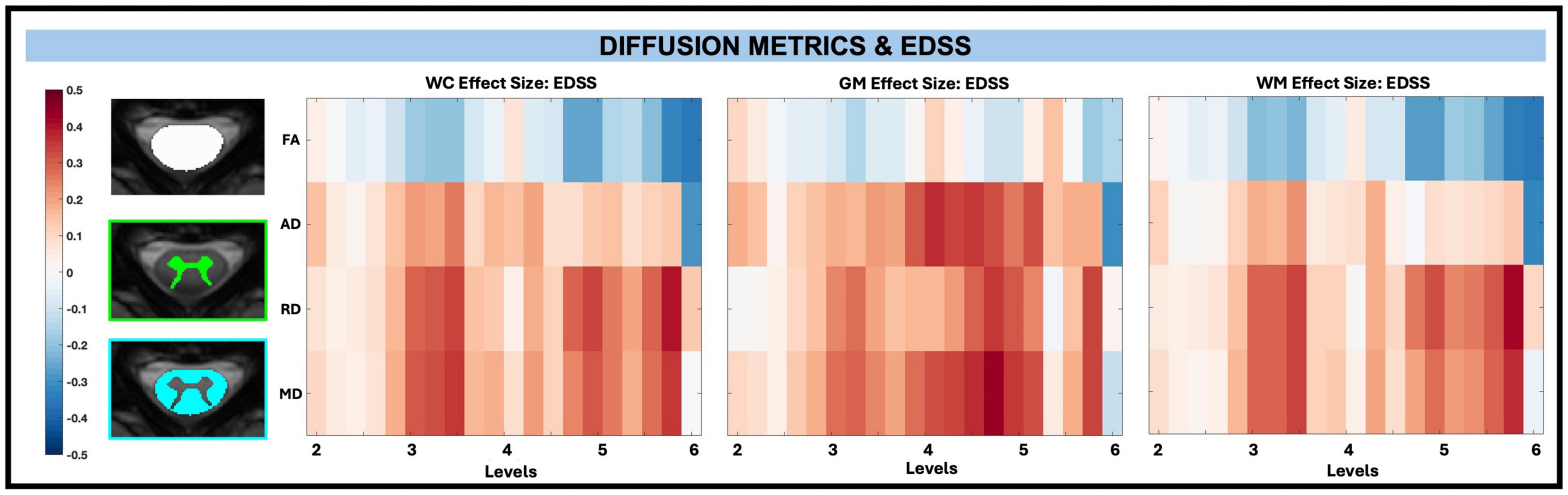
Visualization of linear regression investigating the relationship between diffusion-derived indices (FA, AD, RD, MD) and EDSS for WC, GM, and WM masks. Positive relationships are indicated in red, while negative relationships are indicated in blue.

**Figure 10:**
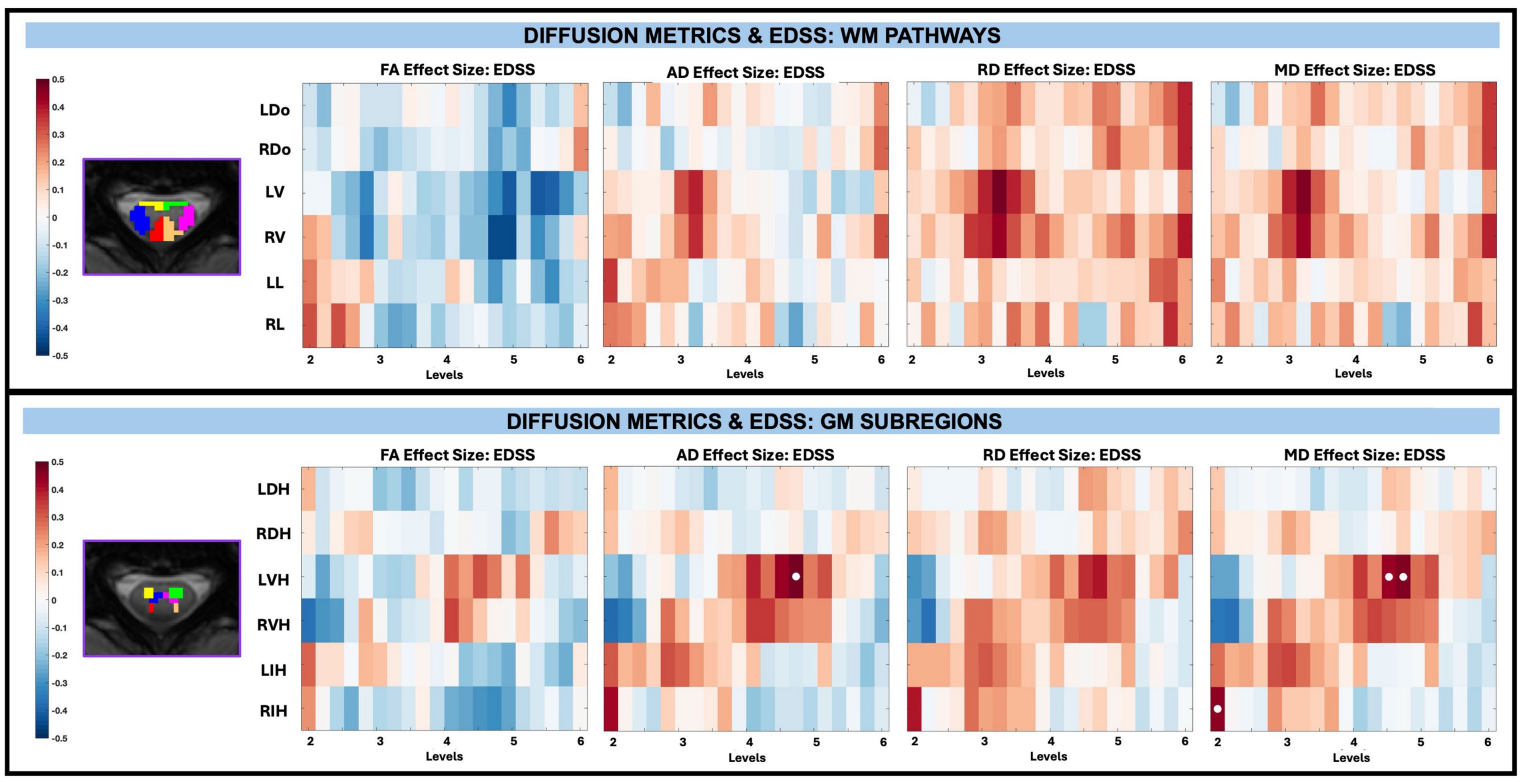
Visualization of linear regression investigating the relationship between diffusion-derived indices (FA, AD, RD, MD) and EDSS for WM pathways (top) and GM subregions (bottom). Positive relationships are indicated in red, while negative relationships are indicated in blue. Significant differences are denoted via a white circle, with *p* < 0.05 following FDR correction.

Across whole cord, WM, and GM masks, diffusion-derived indices (FA, AD, RD, and MD) were not significantly associated with TUG, T25, or EDSS after FDR correction, with EDSS shown in Figure 9. However, significant associations between microstructure (AD and MD) and disability (EDSS) were observed in pwRRMS GM subregions (Figure 10), including positive relationships in GM subregions between EDSS and AD for LVH (1 sub-level), EDSS and MD for LVH (2 sub-levels), and EDSS and MD for RIH (1 sub-level).

In the T25 model after performing FDR correction, there were no significant associations between T25 and diffusion-derived indices in the WM pathways, nor were there significant associations between T25 and diffusion-derived indices in GM subregions. The same was true for TUG and the WM pathways and GM subregions.

## 4 Discussion

This work derived both macrostructural (CSA) and microstructural (FA, AD, RD, MD) measures at multiple spatial scales and precise locations along the SC, enabling a detailed assessment of structural integrity in pwRRMS. We identified discrete differences in microstructural properties between pwRRMS and HCs, as well as associations with EDSS, supporting the potential use of these measures as biomarkers of disease severity.

Taken together, these findings provide a method to capture regional alterations of SC tissue integrity in pwRRMS when compared to HCs. The following sections will explore the implications of these results in the context of previous literature, discuss potential mechanisms underlying these observed changes, and consider the limitations and future directions for this work.

### 4.1 Findings in MS cohort reflect established disability patterns

Consistent with known disease processes and existing literature (Sebastião et al., 2016; Kalinowski et al., 2022; Cohen et al., 2014), our MS cohort with mild disability (EDSS < 4) demonstrated significantly longer mobility times (T25, TUG) compared to the HC cohort. Although pwRRMS were older than HCs, we accounted for this by including age as a covariate in the multivariable linear regression as discussed previously.

### 4.2 No group differences in whole cord macrostructure, but demonstrated group differences in whole cord microstructure

There were no significant differences between whole cord average CSA for pwRRMS compared to HCs. Current literature on CSA as a measure of atrophy in MS is somewhat mixed depending on the patient population. Some studies suggest significantly lower mean CSA as measured at C2 in pwRRMS compared to HCs, with progressive subtypes typically demonstrating greater atrophy (Casserly et al., 2018; Al-Tameemi et al., 2023). Other studies, including a study using this same participant cohort averaged between C3 and C4, do not suggest differences in whole cord or GM atrophy between groups (Combes et al., 2022; Mann et al., 2007). It’s possible the slight increase in whole cord CSA in our patient cohort relates to inflammation and edema and, as suggested previously, may be masking true atrophy (Mann et al., 2007). Alternatively, given our cohort with only mild disability, it’s plausible we are imaging too early in the disease process for significant macrostructural changes to have occurred. Across literature, there is no established consensus for a preferred CSA calculation method, though our results do align with studies applying SCT for CSA calculation like our approach.

Unlike group differences in macrostructure, there were group differences in whole cord microstructure, including significantly decreased FA and AD in pwRRMS compared to HCs. Diminished FA in the cervical SC of pwRRMS has been previously observed in the literature and may indicate a loss of fiber coherence (Combes et al., 2022; Agosta et al., 2008; Valsasina et al., 2005). AD decreases with axonal loss in mouse models of MS (Budde et al., 2009), but cannot discriminate between degrees of axonal injury in humans (Kreiter et al., 2024) and thus clinical correlation may be limited. The subtle increase in RD and MD in pwRRMS compared to HCs are likewise supported by previous work and could also serve as surrogate measures for axonal injury (Naismith et al., 2010). Increased RD is posited to be associated with myelin damage as supported by pathological studies, though FA, AD, RD and MD are inherently nonspecific (Klawiter et al., 2011).

Taken together, our observation of microstructure and macrostructure differences suggests that microstructural abnormalities may arise independently from macrostructural changes and follow different spatial patterns of vulnerability, despite cord atrophy being consistently measured across a wide variety of SC MS studies. It’s believed a loss of axon density and expansion of the extracellular space may contribute to atrophy (Petrova et al., 2018), and would understandably be captured via microstructural alterations like FA even prior to significant macrostructural changes detected via conventional imaging. Additionally, high rates of microstructural damage early in relapsing–remitting disease are associated with development of SC atrophy at 5 years and greater EDSS scores (Gaubert et al., 2025). Our results support this notion, suggesting diffusion imaging may provide additional information prior to the critical point when CSA changes are detectable and significant. A more in-depth longitudinal study would help support this claim and could provide evidence that CSA may not be the most sensitive method to quantify damage early in the MS disease process.

### 4.3 No group differences in WM/GM macrostructure, but demonstrated group differences in WM/GM microstructure

As in the whole cord, there were no significant group differences between WM or GM CSA for pwRRMS compared to HCs, nor for group differences for either the WM pathways and GM subregions.

When considering differences in group-wide WM microstructure, the significantly decreased FA and AD in pwRRMS mirrored the whole cord analysis. This was reflected in the group WM pathway analysis, with significantly decreased FA in the RDo and LV pathways, and significantly decreased AD in the LL pathway. Parcellation of the WM mask into six anatomically relevant pathways provided additional information to determine where in the WM these significant differences exist, though there was no preference for either hemicord (i.e., all differences in the left or right hemicord) nor pathway (i.e., all differences located to the lateral columns).

For GM microstructure, a significant decrease in FA was reflected in the GM subregions as significant differences in the LVH, RVH, and LIH subregions. The significantly decreased FA segregated to the GM ventral horns may provide insight into what regions of the GM butterfly are first to undergo expansion of the extracellular space, or most affected by such edematous processes (Sbardella et al., 2013). While longitudinal exploration would be necessary to support these claims, it’s interesting to consider MS impacts the WM or GM in heterogeneous patterns, with increased susceptibility to damage in specific regions not otherwise captured in the group analysis.

### 4.4 Whole cord, WM, and GM level delineation allows for identification of group differences at specific spatial locations

The general lack of group CSA differences noted in the whole cord, WM, and GM analyses were also noted in the along-level CSA analyses. There were nearly no significant differences between HC and pwRRMS CSA and a trend towards greater whole cord and WM CSA and decreased GM CSA in pwRRMS compared to HCs. Even so, the along-level analyses provided a more in-depth understanding of trends that were otherwise diminished when averaged at the group level.

The subtle increase in CSA from C2 through C5 aligns with known anatomical variation of the cervical SC as it nears the cervical enlargement, as reflected by whole cord and GM along-level CSA. It’s been shown that pwRRMS exhibit SC GM atrophy in the absence of SC WM atrophy, albeit less pronounced than progressive patients, and that SC GM atrophy correlates with disability independent of SC WM atrophy (Schlaeger et al., 2014). Our results support this when querying along the SC at specific levels, with no observed added benefit after dissecting the GM into specific GM subregions. By utilizing an along-level approach, we better locate where spatial differences in macrostructure are most pronounced along the cord and could extrapolate these findings in longitudinal studies to understand the timing and development of such differences.

Furthermore, it’s plausible our separation of vertebral levels into 0.2 segments better identifies alterations in microstructure compared to group values and provides an alternate mechanism to qualitatively describe such differences. Diffusion-derived microstructural metrics may be most sensitive at distinguishing people with RRMS from HCs, as these subtle microstructural differences in the fine-grained along-level approach may reflect early or subclinical tissue injury that is diluted in whole-cord averages.

Though we did not explore whole cord, GM, and WM lesion load beyond quantification per level, lesion load did not fluctuate in any appreciable pattern from C2 to C5. Atrophy – as encompassed by CSA—and lesion load are independent predictors of disability for progressive and relapsing subtypes (Kearney et al., 2015). It’s been shown that, while cortical lesion burden is associated with worsening disability, SC lesion burden is not (Beck et al., 2023). Future work should carefully consider associations between SC lesion load, atrophy, and disability to determine if these findings remain true when considered on a sub-level basis.

### 4.5 WM pathways/GM subregions level delineation allows for identification of group differences at specific spatial locations

While there were no significant differences in WM pathway CSA between pwRRMS and HCs, there was significantly decreased CSA in the LVH GM subregion in pwRRMS not otherwise captured by group-wide differences in GM subregions. More specific analysis of level-dependent changes may provide a more nuanced understanding of macrostructure and atrophy beyond average pathway analysis.

This hierarchical parcellation is most pronounced when comparing group-level differences in microstructure with differences along levels for both the WM pathways and GM subregions. For both WM pathways and GM subregions, the group-wide differences and significant decreases in pwMS FA are expanded upon when using an along-level approach, depicting where exactly along the SC these changes in FA are most pronounced. These changes in FA seem to cluster near the lower cervical SC levels like C4 and C5, which otherwise may have been missed had the group level analysis sufficed. Additionally, for the GM subregions in particular, level-based differences were identified in AD, RD, and MD – including some sub-levels with significantly decreased AD and significantly increased RD or MD in pwRRMS compared to HCs – that were not identified from group differences. Given lower RD of the cervical corticospinal tract at baseline in pwMS is associated with better clinical outcomes after relapse (Freund et al., 2010), it would be worthwhile to localize what levels are most impacted by changes such as this. Our investigation into an along-level approach versus just group-wide differences reveals opportunities to identify these changes, particularly when nulled at the group level.

### 4.6 GM macrostructure changes relate the most to disability metrics

A main goal of this work was to leverage along-level analysis with disability metrics to help piece together where SC pathophysiology may contribute to clinical alterations. The evident reduction in CSA as a proxy measure for atrophy with increasing markers of disability, including increased motor function times and disability scores, has been observed in previous studies of the MS cervical SC (Mann et al., 2007; Schlaeger et al., 2014; Celik et al., 2023; Losseff et al., 1996). Our GM results support the use of C2/C3 vertebral regions to calculate and represent average cervical SC CSA, as performed in prior work, to draw conclusions with disability. These trends did not persist when the analysis was restricted to individual WM pathways and GM subregions, suggesting GM CSA is most sensitive to disability-related changes at a broader anatomic scale rather than at finer levels of parcellation. This finding has potential clinical utility, as it implies that accurate detection of relevant atrophy may not require detailed segmentation of the cord into multiple subregions where an average GM mask may suffice.

### 4.7 Even with along-level differences, microstructure changes do not explain disability

In addition to macrostructure, we explored diffusion-derived indices in the context of disability scores and determined no significant associations between FA, AD, RD, and MD and T25, TUG, and EDSS, respectively, for whole cord, WM, and GM. From previous work in the cervical SC, whole cord diffusion-derived indices do not seem to correlate with EDSS scores, nor with T25 times (Lee et al., 2020; Klineova et al., 2016). However, there is evidence of limited correlations between pathway/subregion and column-based diffusion-derived indices and disability scores. PwMS have lower FA of the lateral cortico-spinal and posterior tracts compared to HCs, and significant associations between EDSS and RD in the lateral cortico-spinal tract and EDSS and RD and FA in posterior columns (Ciccarelli et al., 2007; Naismith et al., 2013). In our work, when separating the analysis based on WM pathways and GM subregions, we likewise found significant associations of increased GM subregion AD and GM subregion MD and increased EDSS. While average cervical cord MD correlates with degree of disability in some studies (Valsasina et al., 2005), our four significant differences are likely not compelling enough to make any one claim about microstructure changes driving disability.

### 4.8 Along-tract parcellation is feasible in the SC, even without explicit fiber tracking

An alternate approach to investigate diffusion along tissue pathways is diffusion tractography, which provides 3D visual representations of fiber groupings with derived metrics along each point in the tract (Lin et al., 2007; Vargas et al., 2008). This often requires segmenting pathways into a predefined number of along-tract points from which to extract metrics. In this manner, tractography can be leveraged to plot at what point differences between patient and HC fiber tracts begin to emerge, specifically when applied for metric comparisons (Pieri et al., 2021). Despite the utility of tractography, deriving WM fiber pathways in the brain and SC of pwMS requires significant post-processing capabilities, with studies of tractography in the MS SC just beginning to emerge (Ciccarelli et al., 2007).

Unlike the brain, the SC and surrounding vertebrae provide anatomically meaningful levels along each fiber pathway that can be used to localize values for comparison across individuals. In this work, we provide an anatomically robust along-tract analysis of the cord, despite not directly utilizing tractography. While our level parcellation is more coarse than intricate fiber tracking, it is more straightforward to apply and requires only a T2 sagittal image or PAM50 atlas to derive vertebral levels instead of specialized software.

### 4.9 Clinical implications

Our results highlight the potential clinical utility of anatomically informed SC imaging, even though longitudinal data are still required to establish the temporal order of macro- and microstructural changes and to verify their prognostic value. First, findings such as ours can help identify spinal levels and tissue compartments most vulnerable to MS-related damage. Second, they underscore the importance of focusing diagnostic imaging protocols on these regions to more accurately assess disease severity. Finally, this framework may inform future therapeutic strategies by guiding the development of targeted interventions aimed at protecting the most affected SC regions early in the disease course, and what tools or metrics (atrophy, microstructural changes) may be best suited as biomarkers in therapeutic efforts.

### 4.10 Limitations and future directions

Limitations in this study include our recruited patient population and analysis methods. Though our MS patient cohort was significantly older than our HC cohort, we accounted for any age-related differences by including age as a covariate in our analyses and believe changes in MS pathophysiology related to age are marginal over the span of 5 years (Wang et al., 2014; Macaron et al., 2023). Additionally, our patient population consisted of individuals with relatively low disability (EDSS < 4), which may have biased our sample towards younger individuals. Future recruitment efforts should aim to include older individuals with more advanced disability to better capture the full spectrum of disease severity. We also recognize that sex differences in MS brain studies are well established, such as more extensive WM damage in males compared to females with MS (Klistorner et al., 2018; Schoonheim et al., 2014). Sex differences in the MS SC are underexplored, and though we considered age and sex as covariates in our statistical analysis, stratifying based on sex within a larger recruited patient population would be of merit to help unravel biologically meaningful sex-related differences in macro- or microstructure at discrete spinal levels.

From an analysis and post-processing standpoint, we are bound to acquisition constraints in the SC (slice thickness 5mm^3^) and thus had to interpolate our cervical levels to better match the PAM50 atlas resolution for CSA calculation. In future iterations, it may be feasible to push our image resolution, though critical to remain mindful of sequence time and participant demand in an MS population. Our selection of WM pathway and GM subregion masks was derived from pre-determined PAM50 atlas masks and may be susceptible to partial volume effects. While we utilized a probabilistic approach, we note the possibility of a single voxel containing information from a mixture of different tissue types or pathways from our 0.5 cutoff.

Finally, we recognize that SC lesion location and distribution in the MS population may contribute to spatial alterations of macrostructure and microstructure. In a post-hoc analysis, we derived diffusion metrics (FA, AD, RD, MD) from normal-appearing WM and GM masks, respectively, and determined broadly similar trends to those included in this manuscript with whole WM and GM masks. Future iterations should include an in-depth analysis of macrostructure and microstructure with specific consideration of lesion burden to more fully describe the regional specificity of SC alterations. We also acknowledge our somewhat limited ability to assess the influence of lesion heterogeneity on diffusion-based findings in particular, given our image acquisition without gadolinium-based contrast. In the brain of pwRRMS, FA values differentiate between different types of MS pathology, including contrast enhancing lesions and iron rim lesions (Shi et al., 2023). If this work is translated to the SC, we may better understand the cellular mechanisms and pathophysiology of MS lesion developmemt and thus offer more specific biomarkers for MS-related treatment strategies.

## 5 Conclusion

This study demonstrates the utility of an along-level diffusion MRI framework for characterizing both macrostructural and microstructural SC abnormalities in pwRRMS. By leveraging the inherent anatomical segmentation of the cervical SC, this approach enables spatially localized detection of disease-related alterations, offering a resolution not achievable with conventional whole-structure averaging. Our results reveal significant group differences in FA and CSA, with spatially distinct patterns of abnormality across WM pathways and GM subregions. Notably, these alterations were often confined to specific cervical levels, underscoring the importance of examining the SC as a functionally and anatomically heterogeneous structure. While GM atrophy exhibited strong associations with clinical disability measures, diffusion-based microstructural changes did not independently explain variance in clinical scores, suggesting a complex and multifactorial relationship between SC damage and functional impairment.

These findings underscore the potential of along-level tractometry as a complementary methodology to tractography, particularly in regions like the SC where anatomical constraints facilitate precise localization. Future work should focus on refining along-level models, integrating multi-parametric imaging, and expanding clinical correlations to further elucidate the biological underpinnings and clinical relevance of localized SC changes in MS.

## Data Availability

All data generated or analyzed during the study are described in Methods section of this paper. We welcome collaboration and access to our de-identified imaging and sensorimotor data as well as the code written to analyze it. To abide by local institutional policies, a signed data transfer agreement will be required for access and download.
